# Minimalist Footwear and Knee Joint Loading During Walking in Healthy Adults: A Systematic Review of Evidence for Osteoarthritis Prevention

**DOI:** 10.3390/jfmk11030372

**Published:** 2026-09-17

**Authors:** Titus Vari, Iulia-Mihaela Vălean, Theodor Popa, Alexandru Roman, Sorana-Carina Buda, Rodica-Ana Ungur, Tudor-Ștefan Ciortea, Viorela-Mihaela Ciortea, Laszlo Irsay

**Affiliations:** 1 2nd Department of Obstetrics and Gynecology, “Iuliu Hațieganu” University of Medicine and Pharmacy, 400610 Cluj-Napoca, Romania; titus.vari@elearn.umfcluj.ro; 2 Department of Rehabilitation Medicine, Clinical Rehabilitation Hospital, 400066 Cluj-Napoca, Romania; iulia.miha.valean@elearn.umfcluj.ro (I.-M.V.); popa.theo94@gmail.com (T.P.); roman.alexandru@elearn.umfcluj.ro (A.R.); buda.soranacarina@elearn.umfcluj.ro (S.-C.B.); rodica.ungur@umfcluj.ro (R.-A.U.); laszlo.irsay@umfcluj.ro (L.I.); 3 Department of Hygiene, “Iuliu Hațieganu” University of Medicine and Pharmacy, 400349 Cluj-Napoca, Romania; 4 Department of Rehabilitation Medicine, “Iuliu Hațieganu” University of Medicine and Pharmacy, Victor Babeș Street 8, 400012 Cluj-Napoca, Romania; 5 Faculty of Medicine, “Iuliu Hațieganu” University of Medicine and Pharmacy, 400347 Cluj-Napoca, Romania; ciortea.tudor.stefan@elearn.umfcluj.ro; 6 Department of Physical Medicine, Balneotherapy and Rehabilitation, “Iuliu Hațieganu” University of Medicine and Pharmacy, 400012 Cluj-Napoca, Romania

**Keywords:** minimalist footwear, barefoot, gait, knee adduction moment, knee osteoarthritis, primary prevention

## Abstract

**Background/Objectives**: Minimalist footwear has been proposed as a passive means of reducing knee loading, but its effects during walking in healthy adults, the population relevant to primary prevention of knee osteoarthritis (KOA), have not previously been synthesized. **Methods**: Following PRISMA 2020 (PROSPERO CRD420261396693), four databases and ClinicalTrials.gov were searched to September 2026 without date restriction. Primary outcomes were peak knee adduction moment (KAM), KAM impulse, peak knee flexion moment (KFM), and patellofemoral contact force; secondary outcomes were vertical loading rate, stride length, cadence, foot strike angle, and foot progression angle as a registered post hoc amendment. Risk of bias was assessed in duplicate with the Cochrane RoB 2 tool for crossover trials; synthesis was narrative; certainty was rated with GRADE. **Results**: Six publications met the criteria, but they derive from only four independent cohorts and 100 unique participants: three of the six report the same cohort of 40, a shared structure disclosed in none of them. Two of the four primary outcomes were reported by a single study in one cohort of 16, which found no difference between minimalist and conventional footwear in either peak KAM or peak KFM; KAM impulse and patellofemoral contact force were not reported by any study. Vertical loading rate, spatiotemporal parameters, and foot progression angle were discordant or non-comparable between cohorts. The meta-analysis was precluded by overlapping samples, absent discrete data and non-comparable outcome definitions; certainty was very low for every outcome. **Conclusions**: The evidence is smaller and less independent than its publication count suggests, and no recommendation for or against minimalist footwear in KOA prevention is currently supportable. Whether these loading markers, studied mainly in relation to progression in established osteoarthritis rather than incident disease in healthy joints, are appropriate prevention targets also remains unclear.

## 1. Introduction

Knee osteoarthritis (KOA) is a major global health burden, with prevalence increasing by more than 130% since 1990 and projected to rise by a further 75% over the next 25 years [1]. It is multifactorial, influenced by age, female sex, obesity, and chronic abnormal joint loading [2,3,4].

Abnormal loading disrupts the homeostasis maintained by chondrocytes and promotes degradation of the extracellular matrix, while subchondral bone remodeling and synovial inflammation accelerate cartilage loss [4,5]. Because these processes are driven by cumulative mechanical stress, joint loading is mechanically modifiable and is therefore of interest as a potential target for intervention.

Three gait-derived loading parameters are most consistently implicated. The knee adduction moment (KAM) is a validated surrogate for medial compartment loading and has been associated with the presence and progression of medial tibiofemoral OA [5,6]. The knee flexion moment (KFM) is strongly related to patellofemoral contact force and contributes to total tibiofemoral loading, though joint kinetics are best predicted when KFM is combined with KAM [7,8,9,10]; its association with longitudinal structural progression is less consistent [6,11]. Vertical loading rate (VLR) reflects the rate of rise of the vertical ground reaction force following foot contact [12,13]. It is, however, an external measure—reflecting body acceleration rather than joint force—and its specific link to knee osteoarthritis remains unestablished [12,14]. Foot progression angle modifies the knee adduction moment and is an active target of gait-retraining interventions in medial knee OA [15,16]; the direction of its effect is peak-specific and highly individual, with personalized targets outperforming uniform toe-in or toe-out prescriptions [17]. These associations derive from populations with established disease, and none of them establishes predictive validity for incident knee osteoarthritis in healthy joints.

Current international guidelines recommend non-pharmacological interventions—low-impact exercise, patient education, and weight management—as the cornerstone of KOA prevention and management [18,19,20]. Long-term adherence, however, remains challenging: first-year dropout rates reach 70% in home-based exercise programs [21,22], and weight-management interventions show limited long-term adherence, with frequent weight regain [23,24]. Interventions that modify joint loading passively, without requiring sustained behavioral change, are therefore of considerable interest.

Minimalist footwear is one such candidate. It is defined by maximal structural flexibility, low mass and stack height, minimal heel-to-toe drop, and the exclusion of motion-control or stability devices [25]. In patients with established KOA, flat and flexible footwear has been reported to reduce the knee adduction moment and to improve pain and function [26,27]. However, these studies evaluated older adults with symptomatic disease using flat-flexible or “mobility” shoes rather than contemporary minimalist footwear, focusing on therapeutic intervention rather than primary prevention.

The existing literature is primarily focused on minimalist running biomechanics and therapeutic applications in patients with established KOA. A recent scoping review mapping minimalist footwear as a clinical intervention across 16 studies identified the exclusion of asymptomatic participants as a major limitation and recommended a systematic review of the available evidence [28].

This systematic review evaluates whether minimalist footwear, compared with conventional footwear, alters knee joint loading markers during walking in healthy, asymptomatic adults. Primary outcomes are markers of medial and patellofemoral compartment loading—peak knee adduction moment, KAM impulse, peak knee flexion moment, and patellofemoral contact force. Secondary outcomes are vertical loading rate, stride length, cadence, foot strike angle, and foot progression angle, with the last added as a registered post hoc amendment after study identification. Additionally, drawing on external validation literature rather than on the included studies, we appraise whether these loading markers have been validated as prognostic in asymptomatic populations, as their clinical use presupposes.

## 2. Materials and Methods

### 2.1. Study Design and Protocol Registration

This review followed the Preferred Reporting Items for Systematic Reviews and Meta-Analyses (PRISMA) 2020 statement [29] and was prospectively registered with the International Prospective Register of Systematic Reviews (PROSPERO; CRD420261396693); the full protocol is accessible via the PROSPERO record. The registered protocol was amended after study identification; the amendments, and the date on which each was registered, are reported in the Limitations.

### 2.2. Eligibility Criteria (PICO Framework)

Studies were selected based on rigid population, intervention, comparison, and outcome (PICO) parameters, designed to isolate primary prevention pathways:

Population (P): inclusion: healthy, asymptomatic adults aged ≥18 years. Exclusion: existing clinical or radiographic knee osteoarthritis, knee pain, neurological conditions affecting gait, previous lower-limb surgery, or structural lower-limb deformity.

Intervention (I): minimalist footwear, defined (insole-inclusive) as follows: zero heel-to-toe drop (≤1 mm); stack height ≤12 mm; high longitudinal and torsional flexibility; and absence of motion-control, stability, or arch-support devices. These criteria were pre-specified in the registered protocol, subject to the stack-height correction described in Section 4.6, which was applied in July 2026 and entered in the registry in September 2026. The drop and stack-height values are operational thresholds adopted for this review, consistent with the consensus definition of minimalist footwear but not derived from a validated cut-off, since the consensus explicitly declined to set one. Where specifications were incomplete, eligibility was determined by the study’s published description, manufacturer specifications, and direct caliper measurements reported in the literature. None of the Ostrava cohort publications reports stack height for either shoe; the minimalist condition in those reports (Vivobarefoot Primus Knit) was therefore classified as eligible on manufacturer specification under this provision. The minimalist index [25] was not used as an eligibility threshold; three publications characterize their footwear by this index rather than by geometry, and the values they report are given in the note to Table 1. High longitudinal and torsional flexibility was specified as a construct criterion but could not be evaluated against any measured value, since no included publication reports a flexibility measurement for any condition tested (note to Table 1); footwear was therefore classified on flexibility using the published descriptions and manufacturer specifications alone.

Comparison (C): Conventional footwear, defined as a cushioned midsole, an elevated heel-to-toe drop of 8 to 12 mm, and a structured upper with a heel counter. Footwear meeting the minimalist specification above is not eligible as a comparator. Fully unshod (barefoot) conditions were excluded. Participants’ own habitual footwear is eligible as a comparator only where that footwear was verified against the stated criteria for conventional cushioned footwear and the verification is reported; habitual or preferred footwear that is not characterized against any stated criteria is not an eligible comparator.

Outcomes (O): Primary outcomes were peak knee adduction moment (KAM), KAM impulse, peak knee flexion moment (KFM), and patellofemoral contact force (PFCF). Secondary outcomes were vertical loading rate (VLR), stride length, cadence, foot strike angle, and foot progression angle, with the last added as a registered post hoc amendment after study identification.

Study design and context: Experimental and quasi-experimental quantitative designs, including randomized and non-randomized crossover trials and longitudinal controlled trials. Excluded: qualitative studies, case reports and series, retrospective reviews, observational cohorts without a controlled footwear intervention, reviews, editorials, conference abstracts, and non-English-language publications.

Setting and instrumentation: Instrumentation requirements were tiered by outcome. Primary outcomes required 3D motion capture with force plates or an instrumented treadmill. Validated pressure platforms or insoles could contribute to secondary outcomes only. Studies relying on visual observation, pedometers, 2D consumer-grade video, or unvalidated inertial measurement units were excluded.

Task: Level, flat walking on laboratory runways or instrumented treadmills. Running protocols, inclined or uneven surfaces, stair negotiation and cutting maneuvers were excluded.

### 2.3. Information Sources and Search Architecture

Four bibliographic databases were searched without chronological restrictions: PubMed, Web of Science Core Collection (Science Citation Index Expanded), Scopus, and the Cochrane Central Register of Controlled Trials (CENTRAL). Clinical trial records were tracked via ClinicalTrials.gov. All databases and the register were last searched on 10 September 2026.

The search strategy differed by source. Scopus was searched on the intervention concept alone, without the knee-loading and locomotion concepts, and the retrieved records were filtered manually at title and abstract. Footwear-biomechanics studies do not reliably carry osteoarthritis or knee-loading terminology in title, abstract or keywords, so a multi-concept Boolean strategy loses eligible reports in this field; searching the broadest-coverage database on the intervention concept alone maximizes sensitivity at the cost of precision. Applying the same single-concept strategy across all four databases was not practicable, since the databases overlap substantially, and the additional yield would have been small relative to the screening burden; Scopus was selected for the broad strategy as the source with the widest coverage. The remaining sources were searched with the full multi-concept strategy combining the intervention concept, the knee-loading concept and the locomotion concept. Intervention terms included both the “minimalist” and “minimalistic” spelling variants. Where a platform did not apply truncation inside quotation marks, wildcards were expanded manually.

Two supplementary methods were used, both pre-specified at registration: backward citation searching of the reference lists of eligible studies and relevant reviews, and AI-assisted literature discovery using the Consensus discovery engine.

The full search strings, field tags, editions and record counts for each source, as executed, are provided in Supplementary Table S1.

Selection process: Records from all sources were deduplicated before screening. Titles and abstracts were then screened independently and in duplicate by two reviewers, blind to each other’s decisions. Records were excluded at this stage only where ineligibility was clear from the title or abstract; records whose task, comparator or outcomes were not stated were carried forward to full-text assessment. Full texts were assessed independently by two reviewers against the same eligibility criteria. Disagreements at either stage were resolved by discussion, with a third reviewer consulted where agreement was not reached.

### 2.4. Data Extraction and Management

Two reviewers extracted data independently and in duplicate using a standardized, pre-piloted template. Any disagreements were resolved by consensus or by consulting a third reviewer. The following information was extracted from each study:

Study profiles: author names, publication year, country of origin, study design (e.g., randomized crossover trials), and laboratory environment details.

Participant metrics: total sample size, biological sex distribution, mean age, and anthropometric data (body mass, height, BMI) confirming healthy, asymptomatic status.

Footwear characteristics: technical validation parameters for the minimalist shoe (sole drop, stack height, and flexibility scores) and the conventional shoe comparator. No included publication reported a flexibility measurement, so this field was extracted but returned no data.

Gait protocol metadata: Walking surface characteristics (ground runway vs. treadmill specifications), gait velocity constraints, capture systems (motion capture brand, force plate models, sampling and filtering frequencies), and participant adaptation states (acute vs. longitudinal).

Biomechanical outcomes: Sample means and standard deviations (SDs) for the primary medial compartment (peak KAM, and KAM impulse) and patellofemoral compartment (peak KFM and PFCF) parameters, alongside secondary spatiotemporal and loading variables (VLR, stride length, cadence, foot strike angle, foot progression angle).

If important kinetic or kinematic data, or subsets of the data, were missing or unclear in the published article, the study authors were contacted directly to request the missing reports or raw datasets.

Sign and axis conventions. This review reports external knee joint moments wherever the source convention permits conversion. Two of the Ostrava cohort publications [30,31] report internal joint moments computed in Visual3D, with positive values denoting extension in the sagittal plane and adduction in the frontal plane. Publication [32] reports knee moments in both planes without stating its sign convention or axis definitions, so values from that report are presented as described in its Results text and no conversion to external moments has been applied. The Methods of the 2024 report state that net internal foot, ankle and knee joint moments were computed in three dimensions using a Cardan rotation sequence and a Newton–Euler inverse dynamics technique [30], and the first author confirmed these conventions and the associated axis definitions in writing (J. Malůš, personal communication, 10 September 2026) [33]; both are consistent with the waveform figures in those publications. Under this convention, the negative frontal-plane values printed in those reports are internal abduction moments and are equivalent to external adduction moments. In the sagittal plane, the absorption-phase value corresponds to an external flexion moment and the propulsion-phase value to an external extension moment; the two phases therefore describe moments acting in opposite directions and are not comparable as a single quantity. Hannigan and Pollard (2021) [34] computed external joint moments by inverse dynamics in the same software, so no conversion was required for that report. Because a moment may be algebraically smaller while being larger in magnitude, this review specifies magnitude explicitly in all comparative statements concerning joint moments.

### 2.5. Risk of Bias Assessment

Two reviewers independently assessed risk of bias in duplicate. Both the Cochrane Risk of Bias 2 (RoB 2) tool for crossover trials and ROBINS-I were registered in the protocol at the outset, and the instrument to be applied was determined by the designs of the studies identified. All included studies evaluated participants across all footwear conditions using randomized within-subject designs, although one [30] additionally incorporated a between-subject grouping factor for minimalist footwear experience and is a mixed rather than a pure crossover design. Methodological quality was therefore assessed exclusively using RoB 2; no unregistered instrument was applied. Because participants cannot be blinded to which footwear they are wearing, studies were not downgraded on this basis provided walking speed was strictly controlled. Two a priori decisions governed the domain ratings. First, in a within-subject crossover in which every participant receives every condition, allocation concealment does not carry the selection bias meaning it has in a parallel-group trial, and the live threat of order and period effects is assessed under D2; D1 was therefore rated low risk wherever footwear order was randomized or counterbalanced, irrespective of whether the sequence-generation method was reported. Second, under D6, high risk was assigned where a publication’s stated conclusions are contradicted by its own reported results, or where significant comparisons present in its figures are absent from its text; some concerns were assigned where outcomes were not pre-registered but primary variables were stated a priori. Disagreements were resolved via consensus.

### 2.6. Certainty of Evidence Assessment

The certainty of evidence for each biomechanical outcome was evaluated using the Grading of Recommendations Assessment, Development, and Evaluation (GRADE) framework adapted for narrative synthesis. Evidence from randomized crossover trials started at high certainty and was downgraded based on five standard criteria: risk of bias, inconsistency, indirectness, imprecision, and publication bias. Imprecision was judged on the number of independent cohorts contributing to an outcome rather than the number of publications, so that non-independent reports of the same cohort do not inflate the apparent precision of an estimate.

### 2.7. Data Synthesis Strategy

Meta-analysis was not performed owing to substantial heterogeneity in study designs, footwear interventions, and outcome measures—differences in footwear characteristics, testing surface (treadmill vs. overground), and analytical framework (discrete peak extraction vs. Statistical Parametric Mapping) that would introduce severe aggregation bias. Results are therefore synthesized narratively using an effect-direction matrix.

Non-independence of overlapping reports. Where two or more reports derive from the same or a substantially overlapping cohort, only one report contributes data to any given outcome. Selection follows a pre-specified hierarchy: first, the report with the most complete data for that outcome, meaning means, standard deviations and paired statistics; second, where completeness is equal, the report whose protocol most closely matches the review’s eligibility criteria; third, where both are equal, the earliest publication. Cohort overlap is judged on author overlap, ethics approval number, recruitment period, sample size and sex distribution, and reported anthropometrics. Where overlap is suspected but cannot be resolved from the published reports, corresponding authors are contacted. All judgements, including suspected but unconfirmed overlap, are tabulated and reported.

Appraisal of prognostic validity. The appraisal of whether the review’s loading markers have been validated as prognostic in asymptomatic populations is a narrative commentary rather than a systematic component of this review: its sources were identified outside the systematic search and were not screened, extracted, assessed for risk of bias, or GRADE-rated. It informs the interpretation of the findings but carries no evidential weight in the review’s conclusions about the effect of minimalist footwear.

## 3. Results

### 3.1. Study Selection

The search retrieved 944 records from the four bibliographic databases (Scopus, *n* = 706; Web of Science, *n* = 105; PubMed, *n* = 78; CENTRAL, *n* = 55) and 19 records from ClinicalTrials.gov. After removal of 213 duplicate records, 750 unique records entered title and abstract screening. Neither supplementary method identified any record not already retrieved by the database searches.

Titles and abstracts were screened independently and in duplicate. Agreement between reviewers was 99.7% (748 of 750 records; Cohen’s κ = 0.86), and the two disagreements were resolved by discussion, with both records carried forward to full-text assessment. Screening excluded 742 records, including all 19 ClinicalTrials.gov records, which failed the eligibility criteria on population, task, intervention or publication type.

**Figure 1:**
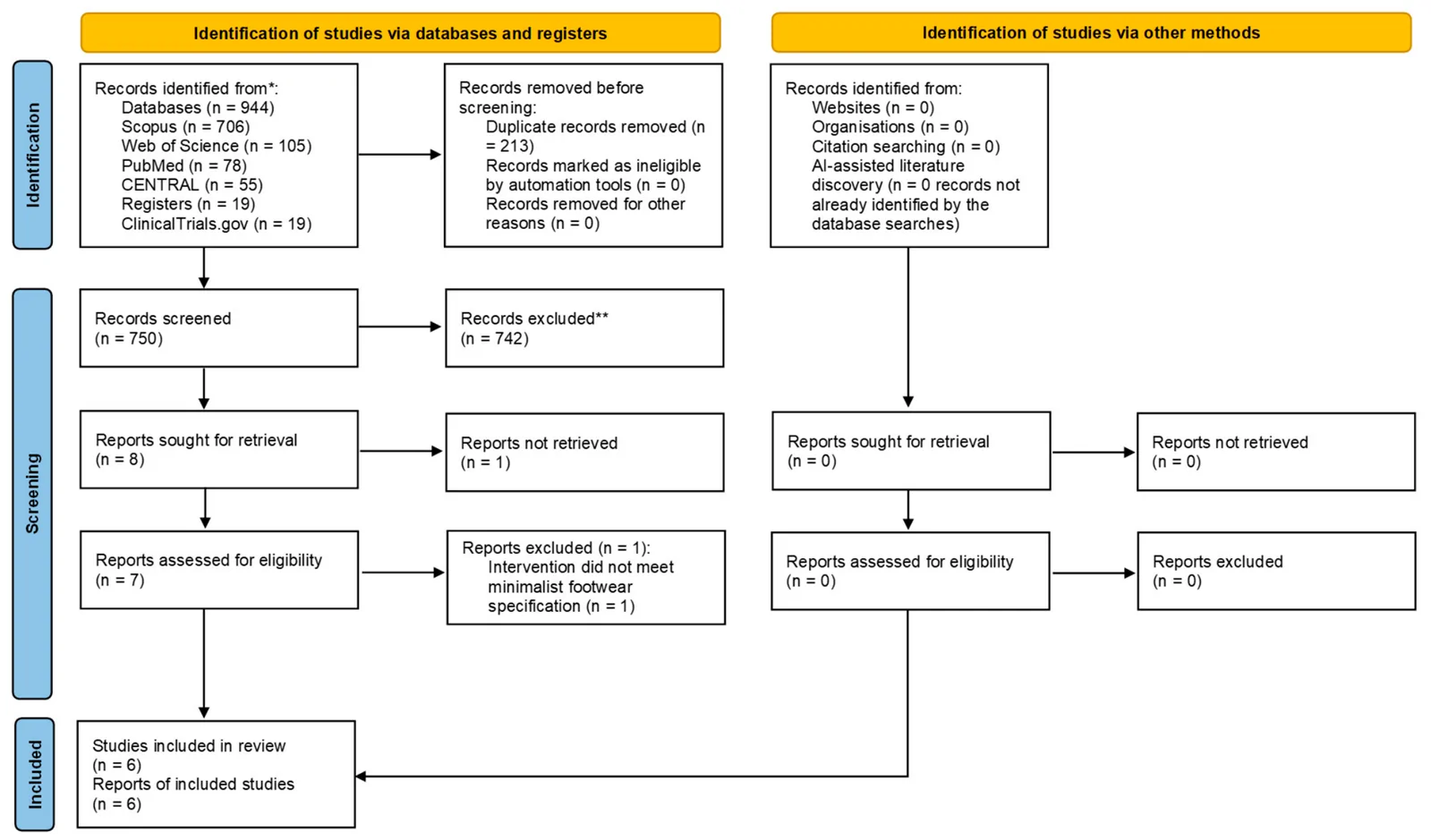
PRISMA 2020 flow diagram of study identification, screening and inclusion. *n*, number of records. * Records identified from each database and register searched, reported separately. ** No automation tools were used; all records were excluded by human screeners.

Eight reports were sought for retrieval, of which seven were obtained. One report (Gulle et al., 2026 [35]) could not be retrieved: no full text was available to us and the corresponding author did not respond to a request. Its abstract indicates that participants were selected for lower-limb structural malalignment (tibial varum ≥ 10°), a population that would not have met the eligibility criteria, but no full-text eligibility assessment was made and the record is therefore reported as not retrieved rather than excluded. Of the seven reports assessed at full text, one was excluded because the intervention did not meet the minimalist footwear specification (Ogaya et al., 2022 [36]; heel-to-toe drop 4 mm, stack height 13 mm, described by its authors as flat-flexible rather than minimalist). Six reports satisfied all eligibility criteria and were included in the narrative synthesis. The PRISMA 2020 flow diagram is presented in Figure 1.

### 3.2. Study Characteristics

**Figure 2:**
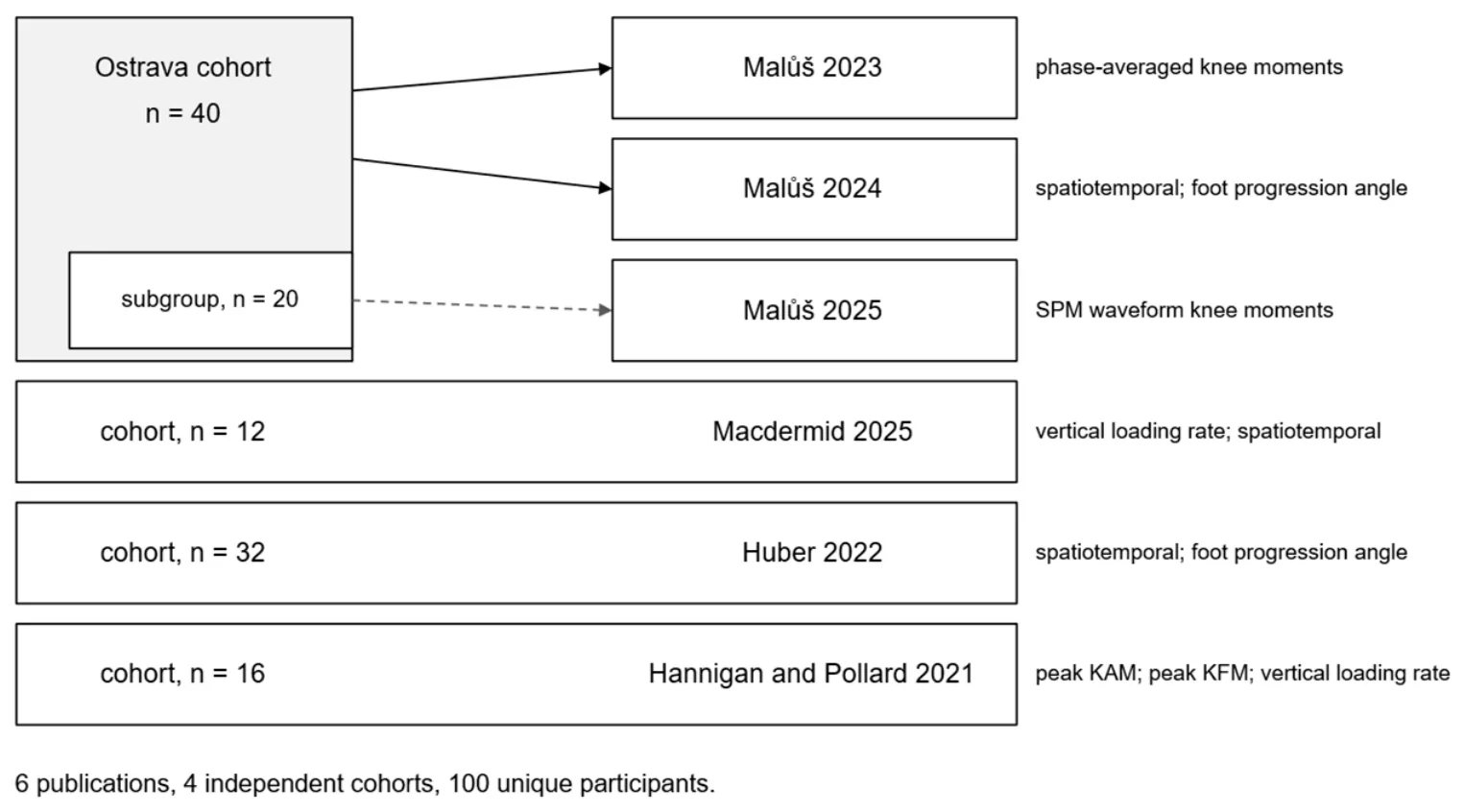
Relationship between the six included publications and the four independent participant cohorts. Three publications derive from a single cohort of 40 healthy adults (Malůš 2023 [31]; Malůš 2024 [30]; Malůš 2025 [32]), one of them analyzing a randomly selected subgroup of 20. The shared-cohort structure is not disclosed in any of the three publications. The remaining three publications each report a separate cohort (Macdermid 2025 [38]; Huber 2022 [39]; Hannigan and Pollard 2021 [34]). Solid arrows indicate analyses of the full 40-participant cohort; the dashed arrow indicates the subgroup analysis (Malůš 2025 [32]). *n*, number of participants; KAM, knee adduction moment; KFM, knee flexion moment; SPM, statistical parametric mapping.

The methodological and demographic characteristics of the six included publications are summarized in Table 1. Together they report a raw total of 160 participants. However, three of them [30,31,32] derive from a single institutional cohort of 40 healthy adults (Figure 2), the 2025 report [32] analyzing a randomly selected subgroup of 20 (J. Malůš, personal communication, 13 July 2026) [37]. The evidence base therefore comprises 100 unique individuals across four independent cohorts. This shared-cohort structure is not disclosed in any of the three publications, and all counts reported in this review are given as publications, independent cohorts and unique participants separately. The four cohorts differ substantially in age and sex composition: three comprise younger adults with a mean age below 40 years, whereas the fourth (Hannigan and Pollard 2021 [34]) comprises 16 women aged 50 to 70 years. That is the age band in which knee osteoarthritis incidence rises most steeply, making it the only cohort in the review approaching the population in which primary prevention would be targeted; but 16 women in a single age band are not representative of healthy adults generally, and the evidence they provide is correspondingly indirect.

**Table 1:** Methodological and demographic characteristics of the included studies.

Study ID	Participants	Tested Footwear Conditions	Protocol and Speed	Instrumentation
Macdermid et al., 2025 [38]	12 F, 24.2 ± 6.2 y; habituated female endurance runners with regular barefoot exposure	Minimalist: 0 mm drop; 5 mm outsole; 206 ± 14 g; durometer 55.0 ± 6.1 HC. Comparator: Asics Gel-Nimbus 25; 8 mm drop; 40.5 mm heel/32.5 mm forefoot; 235 ± 9 g; durometer 30.1 ± 0.1 HC	Treadmill, 1.67 m/s fixed, 1 min (last 10 s logged)	Instrumented treadmill; LoadSol Pro digital pressure insoles
Malůš et al., 2025 [32] ^1,2^	9 M, 11 F, 28.8 ± 5.0 y; sub-sampled recreational athletes	Minimalist: Vivobarefoot Primus Knit. Comparator: Brooks Launch 5	Overground, continuous, 45 min	3D motion capture; Floor-mounted force plates; Pre/post 1.5 T MRI cartilage T2 mapping
Malůš et al., 2024 [30] ^1^	20 M, 20 F, 28.7 ± 4.86 y; recreational athletes (20 minimalist-shoe-experienced, 20 naive)	Minimalist: Vivobarefoot Primus Knit. Comparator: Brooks Launch 5	Overground runway (5 m), 1.45 m/s ± 5% controlled	3D motion capture; Floor-mounted force plates
Malůš et al., 2023 [31] ^1,2^	20 M, 20 F, 28.7 ± 4.86 y; healthy young adults	Minimalist: Vivobarefoot Primus Knit. Comparator: Brooks Launch 5	Overground runway (5 m), 1.45 m/s ± 5% controlled	3D motion capture; Floor-mounted force plates
Huber et al., 2022 [39]	15 M, 17 F, 37 ± 14 y; healthy adults, minimalist-shoe-naive	Minimalist: Leguano city; 0 mm drop; no heel cushioning or arch support; 180 g (EU 39). Comparator: participants’ own daily footwear, verified against published criteria	Treadmill, 0.96 ± 0.16 m/s preferred	Instrumented treadmill; Vertical ground reaction force pressure sensors
Hannigan and Pollard, 2021 [34]	16 F, 59.0 ± 3.6 y; healthy habitual walkers	Minimalist: Merrell Trail Glove; 0 mm drop; 7 mm heel/7 mm forefoot. Comparator: New Balance 880; 12 mm drop; 28 mm heel/16 mm forefoot	Overground, self-selected (1.43 ± 0.19 m/s minimalist; 1.42 ± 0.21 m/s conventional)	3D motion capture (8-camera); Floor-mounted force plates

Note: Data are presented as mean ± SD. M, male; F, female. Extraction was restricted to the minimalist-versus-conventional contrast; barefoot and maximally cushioned conditions were tested by some studies but are not extracted here. Heel-to-toe drop and stack height are reported for both shoes in only two of the six publications, and no publication reports a flexibility measurement for any condition tested. The three Ostrava cohort reports characterize footwear by minimalist index rather than by geometry, all three using the same two shoes (Vivobarefoot Primus Knit, index 96%; Brooks Launch 5, index 28%). Huber et al. used participants’ own habitual footwear as the comparator, verified by the authors against published criteria for conventional cushioned shoes; no geometry is reportable for the comparator. ^1^ Overlapping populations: Malůš [31] and Malůš [30] report the same 40-participant cohort; Malůš [32] reports a 20-participant subgroup of that cohort. The three publications contribute 40 unique participants. ^2^ Anthropometric reporting errors: Malůš [31] reports a height SD of ±70.92 cm and a body mass inconsistent with its own stated BMI; Malůš [32] reports a height SD of ±0.07 cm and gives body mass in kg/m^2^.

### 3.3. Methodological Quality and Risk of Bias

The domain-by-domain evaluation of the six included crossover studies is summarized in Table 2.

Although the RoB 2 assessment flagged all six publications with some concerns regarding unquantified washout periods between footwear conditions, all employed randomized or counterbalanced crossover sequences. Consequently, any acute carryover was likely distributed symmetrically across conditions—inflating variance rather than biasing effect estimates in a consistent direction. Combined with strict speed control and objective instrumented measurement, the overall risk of systematic bias was judged not serious unless outcome-specific reporting concerns justified downgrading.

### 3.4. Certainty of Evidence (GRADE Assessment)

All included studies randomized or counterbalanced the order of footwear conditions and used within-subject designs; baseline certainty was therefore rated high for all outcomes. One study [30] additionally incorporated a between-subject grouping factor for minimalist footwear experience and is a mixed rather than a pure crossover design. The certainty of evidence for each outcome, with reasons for any downgrading, is summarized in Table 3.

Risk-of-bias ratings in Table 3 are outcome-specific rather than derived from each contributing study’s overall RoB 2 label, since a domain concern may bear on one outcome and not another. For peak KAM and peak KFM, the rating is serious because the single contributing study reported twelve outcomes without pre-registration, of which these are two. For vertical loading rate, it is serious because both contributing studies carry the unquantified-washout concern, which bears directly on an acute within-session loading measure. For the Ostrava-derived outcomes it is also serious, but for a more consequential reason: those publications state conclusions that are contradicted by their own reported results. That distinction is recorded here rather than expressed as a further downgrade level.

### 3.5. Narrative Synthesis of Primary Joint Kinetic Outcomes

Two of the four pre-specified primary outcomes, peak KAM and peak KFM, were reported by a single study in a single cohort of 16 participants. KAM impulse and patellofemoral contact force were not reported by any included study. Knee kinetics were reported by three publications from two independent cohorts: Malůš 2023 [31] and Malůš 2025 [32], drawing on the Ostrava cohort, and Hannigan and Pollard 2021 [34]. Malůš 2024 [30] reported knee flexion angles but no knee kinetics, and Huber 2022 [39] and Macdermid 2025 [38] assessed no knee variables. The Ostrava knee-loading evidence rests on one cohort, analyzed twice by two non-comparable approaches (phase averaging and SPM). The only discrete peak moments in the review come from a separate cohort of 16. A supra-threshold interval in statistical parametric mapping indicates where a difference reaches statistical significance across the stance phase, not that the difference is of biomechanical or clinical consequence, and the intervals reported below should be read accordingly.

The studies also differ in walking speed (0.96–1.67 m/s), protocol (treadmill vs. overground), instrumentation and comparator footwear, all of which preclude pooling.

#### 3.5.1. Knee Adduction Moment (KAM)

Peak KAM was reported by one study (Hannigan and Pollard 2021 [34]), which found 0.51 ± 0.16 N·m/kg in minimalist footwear against 0.49 ± 0.14 N·m/kg in the conventional shoe, a difference that was not statistically significant.

Two Ostrava cohort publications reported KAM in other forms. Values in those reports are internal moments and are reported here as external moments (Section 2.4). One [31] reported phase-averaged adduction moment (N·m/kg) and found no significant difference in magnitude between minimalist and conventional footwear in either the absorption phase (internal values −0.20 ± 0.11 vs. −0.19 ± 0.10 N·m/kg, corresponding to external adduction magnitudes of 0.20 and 0.19; *p* = 0.444) or the propulsion phase (−0.18 ± 0.11 vs. −0.20 ± 0.10, magnitudes 0.18 and 0.20; *p* = 0.075). The other [32], analyzing the randomly selected 20-participant subgroup of that cohort, used statistical parametric mapping across the normalized stance phase and reported significantly lower values in minimalist footwear between 0–8% (*p* = 0.022) and 32–90% (*p* < 0.001) of stance, and significantly greater values between 92–100% (*p* = 0.007). That publication did not report its frontal-plane sign convention, so whether these differences correspond to larger or smaller external knee adduction moments cannot be determined from the published report.

These findings are not independent and are only partly reconcilable. Publication [31] presents its frontal-plane data both as discrete phase means in the text and as normalized stance-phase waveforms in Figure 3B of that publication, which comprises mean curves, an SPM{F} main-effect plot and an SPM{t} post hoc plot. The main effect of footwear condition reaches significance only in the final 5% of stance (approximately 95 to 100%, *p* = 0.035). In the post hoc plot, the minimalist-versus-conventional contrast reaches significance in early stance only (approximately 7 to 12% of stance, *p* = 0.0012). The early-stance interval falls within the absorption phase, for which the same study’s discrete phase means show no difference (*p* = 0.444), and it is not reported in the study’s text or addressed in its discussion. The only frontal-plane interval the study reports is the final 5% of stance, whereas the only interval bearing on the minimalist-versus-conventional comparison lies at 7 to 12%; the two do not overlap, which is both why the comparison is absent from the authors’ account and why this review presents it as an observation drawn from the published figure rather than as an established finding.

No estimate of the effect of minimalist footwear on KAM can therefore be derived from the available evidence.

#### 3.5.2. Knee Flexion Moment (KFM)

Peak KFM was reported by one study (Hannigan and Pollard 2021 [34]), which found 0.78 ± 0.36 N·m/kg in minimalist footwear compared with 0.77 ± 0.33 N·m/kg in the conventional shoe (*p* = 0.645).

Two publications reported KFM in other forms. Values in those reports are internal moments and are reported here as external moments (Section 2.4). One [31] reported phase-averaged flexion moment (N·m/kg), with no significant difference between minimalist and conventional footwear in the absorption phase (0.08 ± 0.14 vs. 0.08 ± 0.14; *p* = 0.250), and a significantly smaller-magnitude propulsion-phase moment in minimalist footwear (−0.19 ± 0.10 vs. −0.21 ± 0.11; *p* = 0.001). The other [32], using statistical parametric mapping in that subgroup, reported significantly lower values in minimalist footwear between 3–5% (*p* = 0.026) and 25.5–69.5% of stance (*p* < 0.001), and significantly greater values between 7–19% (*p* < 0.001). As in the frontal plane, that publication did not report its sagittal-plane sign convention, so the direction of these differences in external moment terms cannot be determined from the published report.

The two reports are not independent and differ in analytical resolution (phase averaging vs. continuous waveform analysis). No pooled estimate can be derived from them.

### 3.6. Narrative Synthesis of Secondary Biomechanical Outcomes

Foot strike angle, a pre-specified secondary outcome, was not reported by any included study and is therefore not considered further.

#### 3.6.1. Vertical Loading Rate (VLR)

Two publications from two independent cohorts (28 participants) reported vertical loading rate, with discordant results. Macdermid et al. [38] defined it as the average rate of loading between 20% and 80% of the peak impact force. At the walking condition (1.67 m/s), no significant difference was observed between minimalist and cushioned footwear (14.6 vs. 14.9 N·BW^−1^·s^−1^; *p* > 0.999). Hannigan and Pollard [34] reported significantly higher instantaneous and average vertical loading rates in the minimalist shoe (*p* = 0.001 and *p* = 0.010).

The two estimates are not comparable on population or protocol: Macdermid tested 12 habituated female endurance runners with regular barefoot exposure on a treadmill at a fixed 1.67 m/s using pressure insoles, whereas Hannigan tested 16 habitual walkers of mean age 59 years overground at a self-selected 1.43 m/s using floor-mounted force plates. A significant main effect of footwear on loading rate was present across the full protocol (*p* = 0.028): that study reported significantly lower loading rates in the cushioned shoe at 10 and 14 km/h, but those are running speeds and fall outside this review’s walking eligibility criterion, so only the 6 km/h walking condition was extracted.

#### 3.6.2. Spatiotemporal Adaptations

Cadence: Three studies, from three independent cohorts, reported cadence or its reciprocal. Huber et al. [39] reported a significantly higher cadence in minimalist than in conventional footwear (100.91 ± 6.55 vs. 98.63 ± 7.53 steps·min^−1^; MD 2.28, 95% CI 1.12–3.45; *p* < 0.001; d = 0.89). Malůš et al. [30] found no significant difference (114.15 ± 5.03 vs. 113.88 ± 4.88 steps·min^−1^; *p* = 0.275). Macdermid et al. [38] reported no main effect of footwear on stride duration, the reciprocal of cadence (*p* = 0.144).

Stride/step length: Huber et al. [39] reported a significantly shorter step length in minimalist than in conventional footwear (56.08 ± 7.98 vs. 57.44 ± 7.81 cm; *p* < 0.001). Malůš et al. [30] found no significant difference in stride length (1.48 ± 0.06 vs. 1.51 ± 0.06 m; *p* = 0.209). In Huber’s fixed-speed treadmill protocol, cadence and step length are reciprocally constrained (speed = cadence × step length) and therefore do not represent independent findings.

Stance/ground contact time: Two studies from two cohorts both reported no significant difference: Malůš et al. [30], stance time 0.68 ± 0.31 vs. 0.69 ± 0.03 s (*p* = 0.177); Macdermid et al. [38], no main effect of footwear on ground contact time (*p* = 0.163).

Swing time and time to mid-stance, which, similar to stance and ground contact time, were not pre-specified and are reported here as exploratory observations, were reported by a single study [38]: no footwear effect on swing time (*p* = 0.130); time to mid-stance was significantly shorter in minimalist footwear at the walking condition (0.276 vs. 0.301 s; *p* < 0.001).

Because the three studies used markedly different walking speeds (0.96, 1.45 and 1.67 m/s), a dominant determinant of spatiotemporal parameters, only within-study minimalist-versus-conventional contrasts were extracted.

#### 3.6.3. Foot Progression Angle

Foot progression angle was a post hoc outcome addition. Two studies, from two independent cohorts, reported it.

One study [39] reported a significantly smaller foot progression angle in minimalist than in conventional footwear (6.71 ± 4.12° vs. 7.82 ± 4.26°; MD −1.11°, 95% CI −1.66 to −0.55; *p* < 0.001; d = 0.91), indicating reduced toe-out. A second study [30] reported no difference between the two conditions (−2.13 ± 5.50° vs. −2.11 ± 6.42°; *p* = 1.000).

The two studies reported foot progression angle in opposite sign ranges (Huber positive, 5.07–7.82°; Malůš negative, −1.49 to −2.13°). Huber defined positive as toe-out; Malůš stated no convention. Their values are therefore not directly comparable.

## 4. Discussion

### 4.1. Principal Findings

Two of the four pre-specified primary outcomes have effect estimates, both from Hannigan and Pollard 2021 [34], and both null. KAM impulse and patellofemoral contact force were not reported by any included study. This review therefore provides no evidence that minimalist footwear reduces peak knee adduction or flexion moments during walking in healthy adults.

The one outcome with replication across independent cohorts, vertical loading rate, disagrees between them: no difference at a fixed walking speed in Macdermid 2025 [38], and significantly higher instantaneous and average rates in minimalist footwear in Hannigan and Pollard 2021 [34]. Every remaining outcome rests either on a single cohort or on non-independent reports of the same cohort. Certainty of evidence is very low for every outcome assessed (Table 3), and the main results across all outcomes are summarized in Table 4.

The outcomes examined here are loading markers rather than established risk factors for incident disease, and they have been studied mainly in relation to structural progression in established knee osteoarthritis rather than to the onset of disease in healthy joints.

When the knee moments of the Ostrava cohort were averaged across a phase of the step, minimalist footwear showed no difference in the adduction moment and a smaller-magnitude flexion moment during push-off; a continuous waveform analysis of the 20-participant subgroup reported lower values through the middle-to-late part of the stance phase and greater values in early and very late stance, without stating a sign convention for either plane. In that publication, the abstract describes all three significant adduction-moment windows as reductions, whereas its own Results report the last as an increase. That contradiction lies between two parts of the publication’s own text. The two analyses are not independent. These findings are hypothesis-generating: from a single laboratory and one acute session, no primary-outcome effect estimate can be drawn.

### 4.2. Structure of the Evidence

The shared-cohort structure was confirmed through correspondence with the first author, who indicated that the reports draw on one cohort and “should not be treated as independent samples in a meta-analysis” (J. Malůš, personal communication, 13 July 2026) [37]. Pooling them would count the same 40 participants up to three times. A pre-specified rule therefore restricted each outcome to one report from any overlapping cohort (Section 2.7), and GRADE imprecision was judged on cohorts rather than publications, as shown in Table 3.

### 4.3. Reporting Quality of the Included Studies

During extraction, discrepancies were identified in two of the three Ostrava cohort publications. These discrepancies are of two distinct kinds and are described separately below. Where a discrepancy affected an extracted value, outcome data were taken from the figures and results tables in preference to the abstract or conclusion.

Internal inconsistency within a single publication [32]. Four statements in this publication are contradicted by its own results:

The abstract describes all three significant KAM windows as showing a reduction in minimalist footwear, whereas the Results report the 92–100% window as an increase. Second, the same discrepancy occurs independently in the sagittal plane: the abstract describes all three significant KFM windows as reductions, whereas the Results report the 7–19% window as an increase. Third, the Conclusion states that knee moments increased in all planes in minimalist footwear, whereas the Discussion of the same paper states that the neutral condition showed greater loading between 65 and 100% of stance for the knee adduction moment. Both statements concern which condition was more loaded rather than the sign of a moment, so they are incompatible irrespective of the convention used. Fourth, the Conclusion states that the cartilage T2 response was greater in conventional footwear, whereas both the Results text and Table 4 of that publication report no significant difference between conditions.

The Results and the Discussion also disagree on the extent of the significant KAM interval, reported as 32–90% of stance in the Results and as 65–100% in the Discussion.

Because the abstract and conclusion of this publication are the parts most likely to be read in isolation, and because each discrepancy overstates the definiteness of the finding relative to what that publication’s own results support, we extracted exclusively from the Results text and tables.

Reporting defects without internal inconsistency [31]. The figures and text of this publication agree with one another. Two separate issues nonetheless affect its reporting. The anthropometric data are not internally coherent (see the note to Table 1). Separately, a statistically significant minimalist-versus-conventional difference visible in the post hoc plot of Figure 3B in [31] is neither reported in the text nor addressed in the discussion. Neither issue involves a contradiction between parts of the paper; both concern the completeness and internal plausibility of what is reported. The anthropometric values appear to be transcription errors rather than analytical ones, but whether the erroneous figures propagated into the mass normalization of the reported outcome data cannot be determined from the published reports. The normalized moment and force values extracted in this review are therefore reported as published, and the possibility that they carry a scaling error cannot be excluded. A further defect affects [32]: its sign convention and axis definitions are unstated.

### 4.4. Why Quantitative Synthesis Was Not Possible

Meta-analysis was precluded for four independent reasons, any one of which would have been sufficient:

Non-independence. Three of the six publications derive from a single cohort and cannot be treated as independent samples (Section 2.7).

Insufficient data for the primary outcomes. Peak KAM and peak KFM were each reported by a single study in a single cohort, and KAM impulse and patellofemoral contact force were not reported by any included study. In the Ostrava cohort reports, knee moments were given only as single values averaged over part of the step, or as the segments of the step where a statistical waveform method (SPM) flagged a difference—neither of which provides the means, standard deviations, and paired statistics needed to pool results; the first author confirmed that discrete values were never exported (J. Malůš, personal communication, 13 July 2026) [37].

Outcomes measured in incompatible ways. Variables with the same name were defined and measured differently across studies. Vertical ground-reaction force was reported as a phase-averaged whole-foot mean (N), an anatomically regional peak (N·kg^−1^), and a whole-foot temporal peak (N·BW^−1^)—three distinct quantities that cannot be combined. Foot progression angle was reported using opposite sign conventions without explicit definition. Furthermore, phase-averaged and full-waveform analyses of knee moments are not interchangeable; discrete phase means can mask localized, statistically significant differences within specific sub-windows of the same phase, as observed in one included study.

Protocol and speed heterogeneity. Walking speed ranged from 0.96 to 1.67 m/s across the four cohorts, with two clustered near 1.45 m/s; since speed is the main factor influencing both gait timing and joint forces [40,41], only within-study contrasts could be extracted. This range bears on the spatiotemporal outcomes, for which Huber (0.96 m/s) and Macdermid (1.67 m/s) are the extremes, rather than on the knee-moment comparison: the two cohorts contributing knee moments were tested at a self-selected 1.43 m/s and a controlled 1.45 m/s, respectively, effectively the same speed. Instrumentation (inverse dynamics, floor-mounted force plates, pressure platform, pressure insoles) and comparator footwear (conventional running shoe to cushioned neutral running shoe with a rocker geometry, and in one study participants’ own daily footwear) also differed, and KAM measured in researcher-provided footwear is systematically higher than in participants’ own shoes or barefoot [40]. The discordance is more plausibly driven by speed than by treadmill-versus-overground testing, since the two treadmill studies reached opposite spatiotemporal conclusions.

### 4.5. Findings in Context

The mechanistic rationale for investigating minimalist footwear in prevention is reasonable. Flat, flexible footwear reduces the knee adduction moment and improves pain and function in older adults with established medial knee OA [26,27,42], and the KAM is a modifiable target: a sham-controlled trial of personalized foot-progression-angle retraining reduced KAM and medial knee pain over one year [16]. Both bodies of work used 3D motion capture with force plates and reported discrete peak KAM and KAM impulse. These methods exist and are routinely applied in adjacent fields, and they have now been applied once to the present question: Hannigan and Pollard 2021 [34] used 3D motion capture with force plates to compare minimalist and conventional footwear during walking and found no difference in either peak KAM or peak KFM. That single study, in that cohort and rated very low certainty, constitutes the entire primary-outcome evidence base of this review—a cohort that is not representative of healthy adults generally.

Foot progression angle, a registered post hoc addition to the review’s outcomes, illustrates these interpretive challenges. The two studies reporting it are not directly comparable and the apparent discordance between them cannot be read as a difference in direction (Section 3.6.3). Even setting that aside, the relationship between foot progression angle and knee loading is peak-specific and individual: toe-in tends to reduce the first KAM peak, toe-out the second peak and KAM impulse, and responses vary enough that personalized targets outperform uniform ones [15]. Since neither study measured KAM concurrently, a group-level shift in foot progression angle cannot in any case be read as a directional change in medial loading.

#### Is Reduced Knee Loading the Right Target?

This review, like the studies within it, presumes that reduced knee loading during walking is desirable in healthy individuals. That premise is less secure than it may appear.

In prevalent, established disease, the knee adduction moment is elevated in people with medial knee OA relative to controls in cross-sectional comparison [43] and in meta-regression across 19 gait studies [40]; this establishes association with prevalent disease rather than predictive value for its onset. On incident disease, in one prospective study of asymptomatic older adults, a higher peak KAM showed only non-significant trends toward incident knee pain at 24 months (RR 2.48, 95% CI 0.99–6.20), whereas a higher knee flexion moment was significantly associated with a lower incidence of frequent knee pain (RR 0.25, 95% CI 0.08–0.85) and with lower pain intensity [44]. Consequently, the two moments treated as parallel loading markers in this review may not exhibit parallel behavior within the target population; in those findings, the evidence that a higher KFM is protective was stronger than the evidence that a higher KAM is harmful.

Furthermore, increased loading is not inherently detrimental. In a post-surgical population, patients who developed radiographic OA five years after ACL reconstruction had walked with lower knee adduction moments and medial contact forces early after surgery than those who did not [45]—a pathological adaptation rather than a footwear effect, but one showing that reduced loading is not uniformly protective. Consistent with this, ordinary loading in healthy adults produces only small (0–5%), recoverable changes in cartilage thickness, volume and composition, recovering within about 30 min of walking, on very low certainty evidence [46].

This contextualizes the only cartilage data in the review: 45 min of walking produced significant main effects of loading on T2 relaxation times in both footwear conditions, with no significant difference between them [32]—a normal, recoverable physiological response rather than evidence of differential cartilage stress. We note that the Conclusion of that publication states a greater cartilage response in conventional footwear. That statement is contradicted by the publication’s own Results text and Table 4, both of which report no significant between-condition difference, and the first author has confirmed in writing that the conclusion as published is an error (J. Malůš, personal communication, 14 July 2026) [47]. We have therefore extracted the result reported in the Results and Table 4. The authors themselves list the use of a 1.5 T scanner as a limitation of the T2 acquisition, which we note here because that measurement concern attaches to the cartilage outcome alone rather than to the gait kinetics extracted in this review.

Taken together, these observations raise a more fundamental question than whether minimalist footwear reduces knee moments. The assumption that reduced loading protects a healthy knee depends on treating KAM and KFM as loading markers with prognostic value. But they were validated in individuals with existing osteoarthritis, by tracking whose disease progressed—not in healthy cohorts. In fact, in the one study of healthy older adults, a higher knee flexion moment predicted less future knee pain, not more [44]. The markers may therefore not carry the same meaning in a healthy knee as in a diseased one, indicating that the core question may have been framed too narrowly. Establishing whether minimalist footwear influences osteoarthritis risk will require not only higher-quality studies, but a clearer account of which loading parameters—if any—possess prognostic value in a healthy joint.

One included publication illustrates the same framing problem in miniature. The conclusion in Malůš 2023 [31] that minimalist footwear “could increase the risk of early development of knee OA” is broader than the minimalist-versus-conventional comparisons on which it rests. For that contrast, the study reports no significant difference in peak KAM, no significant difference in propulsion-phase mediolateral force (*p* = 0.102), a propulsion-phase flexion moment of smaller magnitude in minimalist footwear, and an absorption-phase vertical ground reaction force difference of approximately 1% of body weight; the study’s significant mediolateral force result (*p* = 0.001) belongs to the conventional-versus-barefoot contrast, which this review does not extract. The authors themselves note that the corresponding hypothesis was not supported. We do not suggest the conclusion is incorrect. We note that it extends beyond the comparisons reported, and that this review therefore treats it as a hypothesis rather than as a finding.

### 4.6. Limitations

The protocol was amended after study identification and initial extraction had begun. Two amendments affected eligibility: instrumentation requirements were tiered by outcome, allowing validated pressure platforms and insoles to contribute to secondary outcomes only; and the intervention stack-height criterion was corrected, the registered threshold (<5 mm) being unattainable by any commercially available minimalist shoe when measured insole-inclusive. Foot progression angle was added as a secondary outcome, also after study identification; it is labelled as a registered post hoc addition throughout. The amendment dates are as follows: the instrumentation tiering and the foot-progression-angle addition were registered in July 2026, while the stack-height correction was applied from July 2026 and entered in the registry in September 2026. The September 2026 entry formed part of a broader reconciliation of the registration prompted by peer review, in which two further wording discrepancies were also recorded. That same update followed the identification of four defects in the executed search strategy, the search was re-run, screening was repeated in duplicate, and one further study became eligible as a result (PROSPERO CRD420261396693).

Vertical loading rate was a pre-specified secondary outcome, but its link to knee osteoarthritis is weak: it reflects external impact rather than internal joint force, depends on calculation method and speed, and its association with injury is contested even in the running literature where it originated. Its two contributing cohorts also disagree, a discordance accounted for under indirectness in Table 3. Findings on it should not be read as evidence about osteoarthritis risk.

The English-language restriction excluded at least one apparently relevant record. Wang et al. 2021 [48], published in the *Journal of Medical Biomechanics*, appears from its abstract to have met the population, intervention, comparator, task and outcome criteria and reports knee adduction moment, frontal-plane moment arm, angular impulse, peak loading rate, and stride length in ten participants for barefoot, conventional, and minimalist footwear during walking and jogging, using a Vicon system with a Kistler force plate. Given the small number of eligible studies, and that two of the four pre-specified primary outcomes rest on a single study, the exclusion of a report providing KAM and angular impulse is material. Two records in total could not be fully assessed: Wang was excluded at title and abstract screening because it is published in Chinese, and Gulle et al. 2026 [35] could not be retrieved, its likely ineligibility having been inferred from its abstract alone.

Risk-of-bias assessment was constrained by instrument fit: RoB 2, designed for clinical trials, suits within-subject laboratory biomechanics poorly—participants cannot be blinded, carryover and order effects are not directly addressed, and no domain is designed to capture a mismatch between a study’s reported results and its stated conclusions. Two adaptations were therefore made: D1 was rated low risk wherever footwear order was randomized or counterbalanced, irrespective of whether the sequence-generation method was reported, on the grounds that allocation concealment does not carry its parallel-group meaning in a within-subject crossover; D6 was extended so that high risk could be assigned where a publication’s stated conclusions are contradicted by its own reported results.

Two elements of the appraisal of the Ostrava cluster rest on information from the first author—the shared-cohort structure and the absence of exported discrete scalar values—which a reader cannot independently verify, the underlying thesis not being publicly accessible. The remaining points confirmed in correspondence are verifiable in print: the internal-moment convention is stated in the Methods of Malůš 2024 [30], and the error in the 2025 cartilage conclusion is visible in that publication’s own Results text and Table 4.

Finally, the small number of studies precluded funnel-plot assessment of publication bias.

### 4.7. Implications for Research

Determining whether minimalist footwear has a role in the primary prevention of knee osteoarthritis will require studies designed differently from those available.

Footwear characterization. Reporting across this literature is inconsistent in several respects: heel-to-toe drop and stack height are reported for both shoes in only two of the six included publications; three publications characterize footwear by a composite minimalist index instead of by geometry; one uses participants’ own unspecified daily footwear as the comparator; and none reports a flexibility measurement for any condition tested, despite flexibility being definitional to minimalist footwear. One publication reports midsole durometer, which quantifies hardness rather than flexibility, and the minimalist index does not isolate it either: flexibility is one of five equally weighted components, and its developers declined to define a cut-off [25]. Future studies should report heel-to-toe drop, stack height measured insole-inclusive, mass, and a stated flexibility measure for every condition tested. Doing so would allow eligibility in future syntheses to be assessed against a common definition rather than against manufacturer specifications.

Independent cohorts. Because the knee-loading evidence rests on two cohorts, one of which contributes three of the six publications, replication in independently recruited samples remains the most pressing requirement—the more so because the only outcome reported by both cohorts, vertical loading rate, is discordant between them. Shared cohorts must be declared in every report to prevent erroneous pooling.

Discrete outcome reporting. Studies should report peak KAM, KAM impulse, and peak KFM as discrete values with means, standard deviations, and paired statistics, alongside any waveform analysis, so that effects can be pooled; means and standard deviations alone are insufficient, since pooling a within-subject comparison requires the paired statistics.

Standardized and reported walking speed. Speed is a dominant determinant of gait kinetics [41] and exerts the single largest influence on measured KAM—exceeding cohort type or footwear. Walking speed varied widely across the included studies. Future work should nonetheless standardize speed within a defined range (e.g., ~1.3–1.4 m/s [49,50]) or measure and adjust for it.

A defined conventional comparator. Comparator footwear here ranged from a conventional running shoe to a cushioned neutral running shoe with a rocker geometry, and in one study to participants’ own unspecified daily footwear. Since comparator characteristics may influence the magnitude and even direction of any difference, the comparator should be standardized where possible, and its geometry reported on the same terms as the minimalist condition. Where habitual footwear is used, it should be measured rather than described, so that the contrast under test is defined at both ends.

Explicit measurement conventions. Sign conventions and reference axes must be stated for all angular outcomes, and identically named outcomes defined consistently—problems that prevented comparison of foot progression angle and vertical GRF here, and avoidable through reporting alone. Longitudinal designs with structural or clinical outcomes. All current evidence is acute and single-session. Whether any acute change in knee moments translates into a difference in cartilage health or osteoarthritis incidence is untested; longitudinal studies with compositional MRI or clinical endpoints are needed.

Reconsideration of the loading-marker framework. Studies should specify and justify which loading parameters they treat as adverse, and against what evidence in asymptomatic populations, rather than assuming lower loading is protective.

## 5. Conclusions

Peak knee adduction moment and peak knee flexion moment were each reported by a single study in a single cohort of 16 women, with no difference between minimalist and conventional footwear; KAM impulse and patellofemoral contact force were reported by no included study. This review therefore provides no evidence that minimalist footwear reduces peak knee loading during walking in healthy adults, and the certainty of that evidence is very low. The available evidence is insufficient to support any recommendation, for or against, minimalist footwear for the primary prevention of knee osteoarthritis.

The only outcome reported by two independent cohorts, vertical loading rate, disagrees between them. The remaining knee-moment data derive from three publications drawing on a single cohort of 40 adults. The publication reporting continuous waveform differences does not state its sign convention, so the direction of those differences cannot be established from what is published. These observations are hypothesis-generating and cannot be treated as effect estimates.

These loading markers have been studied mainly in relation to structural progression in established knee osteoarthritis, and their meaning in a healthy joint is uncertain: in one prospective study of asymptomatic older adults, a higher knee flexion moment was associated with a lower incidence of frequent knee pain. Separately from the question of what these markers mean, all available evidence is acute and single-session, so whether any difference observed during a single walking trial would translate into a difference in cartilage health or osteoarthritis incidence is untested. Determining whether minimalist footwear has any preventive role will require independently conducted studies that report discrete peak and impulse values with means, standard deviations and paired statistics; state their sign and axis conventions; give full footwear specifications, including heel-to-toe drop, stack height measured insole-inclusive, mass and a stated flexibility measure; and declare cohort overlap where reports share participants. The absence of these elements is what prevented synthesis here, and their presence is what would make adequate powering possible.

## Figures and Tables

**Table 2:** Cochrane RoB 2 risk-of-bias matrix.

Study ID (First Author, Year)	D1: Randomization	D2: Washout/Carryover	D3: Deviations	D4: Missing Data	D5: Measurement	D6: Reporting	Overall
Macdermid et al., 2025 [38]	Low risk	Some concerns	Low risk	Low risk	Low risk	Low risk	Some concerns
Malůš et al., 2025 [32]	Low risk	Some concerns	Low risk	Low risk	Low risk	High risk	High risk
Malůš et al., 2024 [30]	Low risk	Some concerns	Low risk	Low risk	Low risk	High risk	High risk
Malůš et al., 2023 [31]	Low risk	Some concerns	Low risk	Low risk	Low risk	High risk	High risk
Huber et al., 2022 [39]	Low risk	Some concerns	Low risk	Low risk	Low risk	Low risk	Some concerns
Hannigan and Pollard, 2021 [34]	Low risk	Some concerns	Low risk	Low risk	Low risk	Some concerns	Some concerns

D1, randomization process; D2, washout/carryover between footwear conditions; D3, deviations from intended interventions; D4, missing outcome data; D5, measurement of the outcome; D6, selection of the reported result. Cell shading repeats the judgement written in each cell and follows the Cochrane RoB 2 convention: green, low risk of bias; amber, some concerns; pink, high risk of bias.

**Table 3:** GRADE summary of findings matrix.

Biomechanical Outcome	Publications (Cohorts)	Risk of Bias	Inconsistency	Indirectness	Imprecision	Publication Bias	Final Certainty
Peak KAM	1 (1)	Serious	–	Serious	Serious	Not assessable	Very low
Peak KFM	1 (1)	Serious	–	Serious	Serious	Not assessable	Very low
KAM impulse; patellofemoral contact force	0	–	–	–	–	–	No eligible data
Phase-averaged KAM *	1 (1)	Serious	–	Serious	Serious	Not assessable	Very low
Vertical loading rate	2 (2)	Serious	Not serious	Serious	Serious	Not assessable	Very low
Cadence	3 (3)	Serious	Serious	Serious	Serious	Not assessable	Very low
Stride/step length	2 (2)	Serious	Serious	Serious	Serious	Not assessable	Very low
Stance/ground contact time *	2 (2)	Serious	Not serious	Serious	Serious	Not assessable	Very low
Foot progression angle **	2 (2)	Serious	Serious	Serious	Serious	Not assessable	Very low

* Not pre-specified. ** Post hoc addition. In the inconsistency column, an en dash indicates that a single study contributed and inconsistency could not be assessed; “not serious” indicates that two or more studies contributed, and their results were judged consistent, or that their discordance was explained and accounted for under indirectness. Publication bias could not be assessed for any outcome, fewer than the ten studies specified in the protocol having contributed to any single outcome. Cell shading repeats the rating written in each cell: green, not serious; amber, serious; pink, the final certainty rating, which is very low for every assessed outcome. Cells in the judgement columns that are not shaded indicate a domain that was not applicable or could not be assessed, or an outcome with no eligible data.

**Table 4:** Summary of main results.

Outcome	Publications (Cohorts)	Finding
Peak KAM	1 (1)	No significant difference (minimalist 0.51 ± 0.16 vs. conventional 0.49 ± 0.14 N·m/kg) ^1^
Peak KFM	1 (1)	No significant difference (minimalist 0.78 ± 0.36 vs. conventional 0.77 ± 0.33 N·m/kg; *p* = 0.645)
KAM impulse; patellofemoral contact force	0	Not reported by any included study
Phase-averaged KAM	1 (1)	No significant difference in magnitude, absorption, or propulsion
KAM (SPM waveform)	1 (1)	Significantly lower values in MF at 0–8% and 32–90% of stance, greater values at 92–100%; direction not determinable, sign convention unreported
Phase-averaged KFM	1 (1)	No significant difference in absorption; smaller-magnitude propulsion-phase moment in MF
KFM (SPM waveform)	1 (1)	Significantly lower values in MF at 3–5% and 25.5–69.5% of stance, greater values at 7–19%; direction not determinable, sign convention unreported
Vertical loading rate	2 (2)	Discordant: higher under MF in one cohort, no difference in the other
Cadence	3 (3)	Discordant: higher in MF in one study; no difference in two
Stride/step length	2 (2)	Discordant: shorter in MF in one study; no difference in one
Stance/ground contact time *	2 (2)	No difference in either study
Foot progression angle	2 (2)	Reported by two cohorts using opposite sign ranges with no stated convention; values not directly comparable

Note: MF, minimalist footwear. * Not pre-specified. ^1^ The significant omnibus effect for peak KAM in that study lay between the conventional and maximally cushioned conditions; the maximally cushioned arm was not extracted.

## Data Availability

No new data were created or analyzed in this study. All data analyzed derive from the included published studies and their Supplementary Materials; the full search strategy is provided in Supplementary Table S1.

## Supplementary Materials

The following supporting information can be downloaded at: https://www.mdpi.com/article/10.3390/jfmk11030372/s1. Table S1: Search strategy as executed, giving the query syntax, field tags, editions and record counts for each database and register searched, with execution dates; Table S2: PRISMA 2020 checklist.

## Abbreviations

The following abbreviations are used in this manuscript:

2D	Two-dimensional
3D	Three-dimensional
1.5 T MRI	1.5-Tesla magnetic resonance imaging
AI	Artificial intelligence
BMI	Body mass index
BW	Body weight
CENTRAL	Cochrane Central Register of Controlled Trials
CI	Confidence interval
GRADE	Grading of Recommendations Assessment, Development, and Evaluation
HC	Shore C durometer hardness
IMU	Inertial measurement unit
KAM	Knee adduction moment
KFM	Knee flexion moment
KOA	Knee osteoarthritis
MD	Mean difference
MeSH	Medical Subject Headings
MF	Minimalist footwear
MRI	Magnetic resonance imaging
N·m/kg	Newton-metres per kilogram of body mass
OA	Osteoarthritis
PFCF	Patellofemoral contact force
PICO	Population, intervention, comparison, outcome
PRISMA	Preferred Reporting Items for Systematic Reviews and Meta-Analyses
PROSPERO	International Prospective Register of Systematic Reviews
RoB 2	Risk of Bias 2 tool
ROBINS-I	Risk Of Bias In Non-randomized Studies—of Interventions
RR	Relative risk
SD	Standard deviation
SPM	Statistical parametric mapping
TiAb	Title/abstract field tag
TS	Topic search field tag
vGRF	Vertical ground reaction force
VLR	Vertical loading rate
