# Supplementary material for: Minimalist Footwear and Knee Joint Loading During Walking in Healthy Adults: A Systematic Review of Evidence for Osteoarthritis Prevention
Source: J Funct Morphol Kinesiol. 2026 Sep 17;11(3):372. doi: 10.3390/jfmk11030372 (PMC13608185; doi:10.3390/jfmk11030372)
Supplement: Supplementary file 1 [file jfmk-11-00372-s001.zip › Supplementary Table S1.pdf]

Supplementary Table S1. Search strategy as executed, giving the query syntax, field tags, editions and record counts for each database and register searched, with execution dates.

Search strategy as executed	Records retrieved	Date executed
`PubMed: (("minimalist footwear"[tiab] OR "minimalist shoe"[tiab] OR "minimalist shoes"[tiab] OR "minimal footwear"[tiab] OR "minimal shoe"[tiab] OR "minimal shoes"[tiab] OR "barefoot shoe"[tiab] OR "barefoot shoes"[tiab] OR "barefoot footwear"[tiab] OR "zero drop"[tiab] OR "flexible footwear"[tiab] OR "flexible shoe"[tiab] OR "flexible shoes"[tiab] OR "flat shoe"[tiab] OR "flat shoes"[tiab] OR "mobility shoe"[tiab] OR "mobility shoes"[tiab]) OR (minimalistic[tiab] AND (footwear[tiab] OR shoe[tiab] OR shoes[tiab] OR shod[tiab] OR gait[tiab] OR walking[tiab] OR running[tiab] OR barefoot[tiab] OR plantar[tiab] OR foot[tiab]))) AND ("Osteoarthritis, Knee"[MeSH] OR "knee osteoarthritis"[tiab] OR "knee osteoarthrosis"[tiab] OR "gonarthrosis"[tiab] OR "knee adduction moment"[tiab] OR "knee flexion moment"[tiab] OR "knee joint load"[tiab] OR "knee joint loads"[tiab] OR "knee joint loading"[tiab] OR "patellofemoral"[tiab] OR "knee kinetic"[tiab] OR "knee kinetics"[tiab] OR "knee kinematic"[tiab] OR "knee kinematics"[tiab] OR "knee biomechanics"[tiab] OR "vertical loading rate"[tiab] OR "vertical loading rates"[tiab] OR "foot strike"[tiab] OR "stride length"[tiab] OR "cadence"[tiab]) AND ("Walking"[MeSH] OR "Gait"[MeSH] OR walk*[tiab] OR "gait"[tiab] OR ambulat*[tiab])`<br>*PubMed Advanced Search; Title/Abstract and MeSH fields.*	78	10 September 2026
`Web of Science: Core Collection, Science Citation Index Expanded (SCI-EXPANDED), Topic field. Five sets run separately and combined from the search history. Set 1 (intervention phrases): "minimalist footwear" OR "minimalist shoe*" OR "minimal footwear" OR "minimal shoe*" OR "barefoot shoe*" OR "barefoot footwear" OR "zero drop" OR "flexible footwear" OR "flexible shoe*" OR "flat shoe*" OR "mobility shoe*". Set 2 (spelling variant): minimalistic. Set 3 (footwear and locomotion context): footwear OR shoe OR shoes OR shod OR gait OR walking OR running OR barefoot OR plantar OR foot. Set 4 (knee and loading concept): "knee osteoarthritis" OR "knee osteoarthrosis" OR "gonarthrosis" OR "knee adduction moment" OR "knee flexion moment" OR "knee joint load*" OR "patellofemoral" OR "knee kinetic*" OR "knee kinematic*" OR "knee biomechanic*" OR "vertical loading rate*" OR "foot strike" OR "stride`	105	10 September 2026

length" OR "cadence". Set 5 (locomotion concept): walk* OR gait OR ambulat*. Combination: (#1 OR (#2 AND #3)) AND #4 AND #5		
Scopus: searched on the intervention concept alone and filtered manually at title and abstract. TITLE-ABS-KEY("minimalist footwear" OR "minimalist shoe*" OR "minimal footwear" OR "minimal shoe*" OR "barefoot shoe*" OR "barefoot footwear" OR "zero drop" OR "flexible footwear" OR "flexible shoe*" OR "flat shoe*" OR "mobility shoe*") OR (TITLE-ABS-KEY(minimalistic) AND TITLE-ABS-KEY(footwear OR shoe OR shoes OR shod OR gait OR walking OR running OR barefoot OR plantar OR foot)) *Scopus Advanced Search; TITLE-ABS-KEY field.*	706	10 September 2026
CENTRAL (Cochrane Library, Cochrane Central Register of Controlled Trials, Search Manager): (("minimalist footwear" OR "minimalist shoe" OR "minimalist shoes" OR "minimal footwear" OR "minimal shoe" OR "minimal shoes" OR "barefoot shoe" OR "barefoot shoes" OR "barefoot footwear" OR "zero drop" OR "flexible footwear" OR "flexible shoe" OR "flexible shoes" OR "flat shoe" OR "flat shoes" OR "mobility shoe" OR "mobility shoes"):ti,ab,kw OR ((minimalistic):ti,ab,kw AND (footwear OR shoe OR shoes OR shod OR gait OR walking OR running OR barefoot OR plantar OR foot):ti,ab,kw)) AND ([mh "Osteoarthritis, Knee"] OR "knee osteoarthritis" OR "knee osteoarthrosis" OR "gonarthrosis" OR "knee adduction moment" OR "knee flexion moment" OR "knee joint load" OR "knee joint loads" OR "knee joint loading" OR "patellofemoral" OR "knee kinetic" OR "knee kinetics" OR "knee kinematic" OR "knee kinematics" OR "knee biomechanic" OR "knee biomechanics" OR "vertical loading rate" OR "vertical loading rates" OR "foot strike" OR "stride length" OR "cadence"):ti,ab,kw AND ([mh "Walking"] OR [mh "Gait"] OR walk* OR "gait" OR ambulat*):ti,ab,kw *Cochrane Library, CENTRAL, Search Manager; title, abstract, keyword and MeSH fields.*	55	10 September 2026
ClinicalTrials.gov: Intervention/Treatment field: "minimalist footwear" OR "minimalistic footwear" OR "minimalist shoes" OR "minimalistic shoes" OR "minimal footwear" OR "minimal shoes" OR "barefoot shoes" OR "barefoot footwear" OR "zero drop" OR "flexible footwear" OR "flexible shoes"; Other Terms field: "knee osteoarthritis" OR "knee adduction moment" OR "joint loading" OR biomechanics OR walking *ClinicalTrials.gov search form; Intervention/Treatment and Other Terms fields.*	19	10 September 2026
**Total identified**	**963**	

Records from the four bibliographic databases were deduplicated before screening; of 944 records, 213 duplicates were removed, leaving 731 unique records for title and abstract screening. The 19 register records were not part of this deduplication and were screened alongside the unique database records, giving 750 records screened in total. Terms retained in the strategy as executed that return no records are not removed. PubMed reported that three quoted phrases ("barefoot footwear", "barefoot shoe", "knee biomechanic") are absent from its phrase index and contribute no records.

Scopus was searched on the intervention concept alone, with manual filtering at title and abstract; the remaining sources were searched with the full multi-concept strategy. The rationale is given in Methods 2.3.
